# Correction to: AC016405.3, a novel long noncoding RNA, acts as a tumor suppressor through modulation of TET2 by microRNA‐19a‐5p sponging in glioblastoma

**DOI:** 10.1111/cas.16277

**Published:** 2024-08-09

Siyang Ren, Yinghui Xu. AC016405.3, a novel long noncoding RNA, acts as a tumor suppressor through modulation of TET2 by microRNA‐19a‐5p sponging in glioblastoma. Cancer Science. 2019;110:1621–1632.

In Siyang Ren and Yinghui Xu (2019), Figures 3I and 3J were published with incorrect images. Figure 3 has been replaced by the correct image shown below.

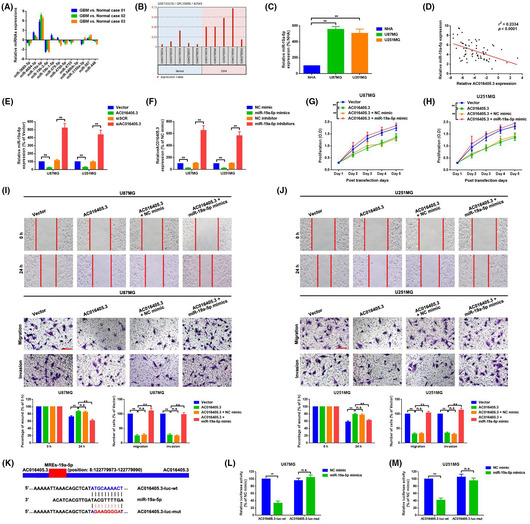

We apologize for this error.

